# Tissue S100/calgranulin expression and blood neutrophil-to-lymphocyte ratio (NLR) in dogs with lower urinary tract urothelial carcinoma

**DOI:** 10.1186/s12917-022-03513-z

**Published:** 2022-11-21

**Authors:** Jana Weinekötter, Corinne Gurtner, Martina Protschka, Wolf von Bomhard, Denny Böttcher, Annika Schlinke, Gottfried Alber, Sarah Rösch, Joerg M. Steiner, Johannes Seeger, Gerhard U. Oechtering, Romy M. Heilmann

**Affiliations:** 1 Department for Small Animals, College of Veterinary Medicine, Leipzig University, An den Tierkliniken 23, 04103 Leipzig, SN Germany; 2 Institute of Animal Pathology, Department of Infectious Diseases and Pathobiology, Vetsuisse Faculty, University of Bern, Länggassstrasse 122, CH-3001 Bern, BE Switzerland; 3 Institute of Immunology, College of Veterinary Medicine, Biotechnological-Biomedical Center, Leipzig University, Deutscher Platz 5, 04103 Leipzig, SN Germany; 4 Specialty Center for Veterinary Pathology, Hartelstrasse 30, E80689 Munich, BY Germany; 5 Institute for Veterinary Pathology, College of Veterinary Medicine, Leipzig University, An Den Tierkliniken 33, E04103 Leipzig, SN Germany; 6 Small Animal Clinic, University of Veterinary Medicine Hannover Foundation, Bünteweg 9, 30559 Hannover, NI Germany; 7 Gastrointestinal Laboratory, Department of Small Animal Clinical Sciences, College of Veterinary Medicine and Biomedical Sciences, Texas A&M University, TAMU 4474, College Station, TX 77843-4474 USA; 8 Institute of Anatomy, Histology and Embryology, College of Veterinary Medicine, Leipzig University, An den Tierkliniken 43, 04103 Leipzig, SN Germany

**Keywords:** Biomarker, Calprotectin, Cancer, Inflammation, S100A8/A9, S100A12, Transitional cell carcinoma, Tumor immunology, Urinary tract infection, Urolithiasis

## Abstract

**Background:**

Urothelial carcinoma (UC) is the most common neoplasm of the canine lower urinary tract, affecting approximately 2% of dogs. Elderly female patients of certain breeds are predisposed, and clinical signs of UC can easily be confused with urinary tract infection or urolithiasis. Diagnosis and treatment are challenging given the lack of disease-specific markers and treatments. The S100A8/A9 complex and S100A12 protein are Ca^2+^-binding proteins expressed by cells of the innate immune system and have shown promise as urinary screening markers for UC. The neutrophil-to-lymphocyte ratio (NLR) can also aid in distinguishing certain neoplastic from inflammatory conditions. Our study aimed to evaluate the tissue expression of S100/calgranulins and the blood NLR in dogs with UC. Urinary bladder and/or urethral tissue samples from dogs with UC (*n* = 10), non-neoplastic inflammatory lesions (NNUTD; *n* = 6), and no histologic changes (*n* = 11) were evaluated using immunohistochemistry. Blood NLRs were analyzed in dogs with UC (*n* = 22) or NNUTD (*n* = 26).

**Results:**

Tissue S100A12-positive cell counts were significantly higher in dogs with lower urinary tract disease than healthy controls (*P* = 0.0267 for UC, *P* = 0.0049 for NNUTD), with no significant difference between UC and NNUTD patients. Tissue S100A8/A9-positivity appeared to be higher with NNUTD than UC, but this difference did not reach statistical significance. The S100A8/A9^+^-to-S100A12^+^ ratio was significantly decreased in neoplastic and inflamed lower urinary tract tissue compared to histologically normal specimens (*P* = 0.0062 for UC, *P* = 0.0030 for NNUTD). NLRs were significantly higher in dogs with UC than in dogs with NNUTD, and a cut-off NLR of ≤ 2.83 distinguished UC from NNUTD with 41% sensitivity and 100% specificity. Higher NLRs were also associated with a poor overall survival time (*P* = 0.0417).

**Conclusions:**

These results confirm that the S100/calgranulins play a role in the immune response to inflammatory and neoplastic lower urinary tract diseases in dogs, but the tissue expression of these proteins appears to differ from their concentrations reported in urine samples. Further investigations of the S100/calgranulin pathways in UC and their potential as diagnostic or prognostic tools and potential therapeutic targets are warranted. The NLR as a routinely available marker might be a useful surrogate to distinguish UC from inflammatory conditions.

**Supplementary Information:**

The online version contains supplementary material available at 10.1186/s12917-022-03513-z.

## Background

Urothelial carcinoma (UC), previously referred to as urinary tract transitional cell carcinoma (TCC), is an epithelial neoplasm originating from the urothelium. It is the most common neoplasia affecting the lower urinary tract (i.e., bladder and urethra) in dogs and comprises approximately 2% of all malignancies in this species [1–3]. Affected dogs are typically elderly females of small- to medium-size breeds. Other known risk factors for UC development include obesity, certain breed predispositions (e.g., Scottish terrier), and chronic exposure to herbicides and pesticides [4–6]. In contrast, vegetable consumption appears to have a protective effect [7].

Clinical signs are usually nonspecific and include hematuria, stranguria, and pollakiuria [1, 3, 8–10]. These may result from the effects of the tumor or a complicating bacterial urinary tract infection (UTI), a frequent concurrent diagnosis in UC patients [11]. Clinical signs in UC patients often do not occur until the late stages of the disease and can be confused with a UTI, rendering UC diagnosis challenging in clinical practice. With metastatic disease, lameness or neurological deficiencies may be seen if bone metastasis occurs to the lumbar vertebrae, pelvis, or limbs [12–14]. In patients suspected of UC, diagnostic imaging (abdominal ultrasonography and/or computed tomography) aids in patient evaluation [15] as UC typically reveals a papillary growth pattern [1, 3]. A single location (typically the trigonal area of the urinary bladder) can be affected in some cases, but often UC is multifocal due to the “field effect” of urine [16]. A minimal database is recommended in all dogs, but urine should not be obtained by cystocentesis due to the risk of needle tract implantation (“seeding effect”) [17]. Several treatment options have been reported with varying outcomes [18–25]. The best overall survival times are achieved by combining surgical and medical treatment options comprised of cyclooxygenase (COX)-2 inhibitors and chemotherapy [8, 19, 26–28]. Many chemotherapeutics have been studied [22, 29, 30], but UC treatment remains challenging as there is no gold standard treatment protocol, and further research is needed to identify more effective therapeutic targets [1, 3, 9, 21, 31].

Different diagnostic modalities for UC diagnosis vary in specificity and sensitivity. In only 30% of affected dogs, tumor cells can be detected on urine sediment analysis. Also, it can be very challenging to distinguish neoplastic from highly reactive urothelial cells [12, 13]. The urine veterinary bladder tumor antigen (V-BTA) test is highly sensitive, easily performed, and non-invasive, but has a low specificity to distinguish UC from UTI, particularly in dogs with glucosuria, hematuria, pyuria, or proteinuria [32]. Although a recently developed diagnostic test for the *BRAF* mutation has a higher diagnostic accuracy [33–36], histopathologic examination of tissue biopsy samples remains the gold standard diagnostic. Sampling such biopsies usually requires cystoscopic examination [37]. Thus, screening tests for dogs at risk would be highly favorable to spare dogs that are not-affected from general anesthesia. While novel diagnostic tools would be favorable, prognostic markers and effective treatment options are also lacking. Few studies have evaluated pathway-specific therapeutic targets (beyond COX-inhibition), including the tyrosine kinase inhibitors lapatinib [38] and toceranib [39]. Further evaluating cell signaling pathways in UC appears to be a promising approach to exploring new ways to modulate molecular signals therapeutically.

S100A8/A9 (calprotectin or calgranulin A/B complex) and S100A12 (calgranulin C) belong to the family of Ca^2+^-binding S100 proteins of the innate immune system [40]. These alarmins play an important role in several neoplastic and inflammatory diseases in humans and dogs and are valuable diagnostic and potentially prognostic biomarkers in human medicine [40–48]. However, their role in UC pathogenesis and their diagnostic use in veterinary medicine is currently limited. A recent study suggested measuring the S100/calgranulins in urine samples to be a promising biomarker for distinguishing inflammatory from neoplastic urinary tract conditions [49, 50]. Higher concentrations of the S100/calgranulins were detected in dogs with inflammatory or neoplastic urinary tract diseases than in healthy dogs, and the S100A8/A9-to-S100A12 ratio was significantly lower in dogs with inflammatory conditions compared to those with neoplastic disease or health [49]. However, the source of S100/calgranulin expression in these conditions remains unknown, and to the authors’ knowledge, S100A8/A9 and S100A12 have not been evaluated in urinary bladder and urethral tissue samples from dogs. Thus, this study aimed to compare the expression of the S100/calgranulins in biopsy samples of neoplastic and inflammatory lesions in urinary bladder and urethral tissue using immunohistochemistry (IHC). We hypothesized that the individual expression and ratio of the S100/calgranulins in lower urinary tract tissue samples mirror the urinary concentrations previously reported for dogs with these conditions. A secondary aim was to evaluate whether S100/calgranulin expression in neoplastic tissue is limited to innate immune cells (granulocytes) or if neoplastic cells also express these proteins.

S100/calgranulin proteins are predominantly produced and released by granulocytes (but also monocytes and macrophages) in the intestinal mucosa. This local innate immune response might extend systemically and be reflected by systemic leukocyte differences, which have not been evaluated in canine UC. The neutrophil-to-lymphocyte ratio (NLR) is an inexpensive marker derived from the minimum database. Given the interest in new inexpensive and simple predictive tools, the blood NLR has been shown to have diagnostic and/or prognostic potential in human patients with inflammatory and neoplastic diseases, including UC [51–54]. Consequently, great interest in the NLR as a biomarker arose in veterinary medicine, where the NLR shows promise as an indicator of outcome in several inflammatory [55–58] and neoplastic [59, 60] conditions. These include chronic inflammatory enteropathy [56], pneumonia [57], acute diarrhea in puppies [58], soft tissue sarcoma [59], and multi-centric lymphoma [60]. Because the NLR has not been studied in canine UC, the third aim of the study was to evaluate the NLR in dogs with UC and their potential to differentiate affected dogs from those with NNUTD.

## Results

### Study population

*Dogs with UC* – Breeds included Airedale terrier, Bernese Mountain dog, Bordeaux mastiff, Border collie, Cocker spaniel, Eurasian dog, Jack Russell terrier, Soft Coated Wheaton terrier, West Highland White terrier (each *n* = 1), Labrador retriever (*n* = 3), and mixed breed dogs (*n* = 11) (Suppl. Table 1). The UC group comprised predominantly female patients (82%), of which 6 dogs (33%) were spayed. UC was confirmed by histopathological examination in all urinary tract tissue biopsies. A urine culture was performed in 12 of the 22 dogs, 3 (25%) of which were positive for bacterial growth (*Escherichia coli*: *n* = 2; *Staphylococcus intermedius*: *n* = 1). Treatment at the time of tissue biopsy included non-steroidal anti-inflammatory drugs (NSAID; carprofen: *n* = 1; firocoxib: *n* = 1; meloxicam: *n* = 11; robenacoxib: *n* = 1).

Sufficient tissue biopsy material for IHC analyses was available from 9 dogs diagnosed with UC (Table 1). The most common tumor location was the urinary bladder (*n* = 6), followed by the urethra (*n* = 4). All tissue biopsies from patients that survived to discharge from the hospital (*n* = 8) were obtained via urethrocystoscopy guidance. One tissue biopsy was taken during necropsy, and pulmonary metastasis was confirmed post-mortem in this patient. Three dogs were suspected or confirmed to have metastases or another neoplastic process in the spleen (*n* = 2), liver (*n* = 1), uterus (*n* = 1), mammary gland (*n* = 1), and/or lung (*n* = 1) based on diagnostic imaging. None of these sites were sampled.

**Table 1:** Patient characteristics of all dogs included in the study (*n* = 55)

Parameter	Urothelial carcinoma	Non-neoplastic urinary tract disease	Healthy control dogs	*P* value^†^
Total number of dogs	22	26	7	–
analyzed by IHC	9	5	6	
in NLR analysis	21	26	1	
Age^†,*^, in years	**11.2**^**A**^	**6.6**^**B**^	**6.5**^a,^^**B**^	**0.0001**
median [range]	[6.8–14.3]	[0.3–14.5]	[2.0–10.0]	
Weight, in kg	26.0	14.5	21.8	0.5074
median [range]	[6.2–49.0]	[3.0–54.0]	[5.2–41.5]	
Sex				
male (n) / female (sp)	4 (2) / 18 (6)	20 (5) / 6 (4)	7 (2) /0 (0)	**< 0.0001**
Breed				
pure-bred	12 (55%)	21 (81%)	5 (71%)	0.1450
mixed breed	10 (45%)	5 (19%)	2 (29%)	
Biopsy type^b^				
endoscopic	9 (90%)	3 (43%)	1 (14%)	–
surgical	0	4 (57%)	0	
post-mortem	1 (10%)	0	6 (86%)	
Primary disease location^b^				
urethra	4 (40%)	1 (20%)	–	–
urinary bladder	6 (60%)	4 (80%)		
Tissues evaluated^b^				
urethra	5 (50%)	1 (17%)	5 (71%)	–
urinary bladder	6 (60%)	6 (83%)	7 (100%)	
Treatment				
treatment-naïve	8 (36%)	19 (79%)^c^	–	–
NSAID treatment	14 (64%)	5 (21%)^c^		
Urine culture				
positive	3 (25%)^d^	8 (44%)^e^	–	–
negative	9 (75%)^d^	10 (56%)^e^		

*IHC* Immunohistochemistry, *n* Neutered, *NLR* Neutrophil-to-lymphocyte ratio, *NSAID* Non-steroidal anti-inflammatory drug, *sp* Spayed

^†^bold font indicates significance at *P* < 0.05

^* ^values with a different capital superscript letter (A, B) differ significantly at *P* < 0.05

^a ^only available from 6 control dogs

^b ^only shown for dogs included in IHC analyses

^c ^only available from 24 dogs

^d ^only available from 12 dogs

^e ^only available from 18 dogs

Follow-up information was available from 18 dogs. At the end of the study, 3 dogs were still alive with stable disease for up to 575 days under mitoxantrone chemotherapy (*n* = 1; 192 days) or NSAID treatment (*n* = 2; 179 and 575 days). All remaining dogs (*n* = 15) were euthanized either immediately after diagnosis (*n* = 3) or within 2–330 days (median: 16 days) of diagnosis, while one dog was diagnosed with UC on necropsy. All but 3 dogs received NSAID treatment, one dog received a combination of meloxicam and metronomic chlorambucil chemotherapy and lived another 180 days, and one dog that received chemotherapy with mitoxantrone was euthanized 25 days after diagnosis. The dog with a 330 day-survival was treated with NSAID and alfuzosin.

*Dogs with non-neoplastic urinary tract inflammation* – Breeds included Yorkshire terrier (*n* = 4), Labrador retriever, Schnauzer (each *n* = 2), Beagle, Bernese Mountain dog, Berger de Brie, Chihuahua, Dachshund, Dalmatian, Doberman, English bulldog, German Shepherd dog, Newfoundland, Pug, Rottweiler, Shih tzu (each *n* = 1), and mixed breed dogs (*n* = 5) (Suppl. Table 1). Diseases included urolithiasis (*n* = 22) and/or histologically confirmed cystitis/urethritis (*n* = 7). Urine culture was performed in 18/26 dogs and was positive for bacterial growth in 8/18 (44%) dogs, and a negative culture was seen in the remaining 10 dogs. Bacterial isolates were *S. intermedius* (*n* = 5), *Proteus mirabilis* (*n* = 1), *E. coli* (*n* = 1), *Streptococcus canis* (*n* = 1), and *Enterobacter cloacae* (*n* = 1); one dog had a polymicrobial urinary tract infection. Most dogs in this group were male (*n* = 20; 77%), five of which (25%) were neutered. The remaining dogs (*n* = 6; 23%) were females.

Follow-up information was available from 17 dogs. Ten dogs were still alive at the conclusion of the study, one dog with recurrent urolithiasis-associated micturition disorder resolved by catheterization. Nine dogs were lost to follow-up. The remaining 7 dogs were euthanized (*n* = 6; within 2–1,247 days after diagnosis, median: 106 days) or had died (*n* = 1; after 4 days); one dog surviving 90 days was euthanized for reasons unrelated to the urinary tract disease.

Sufficient tissue material for IHC staining and analysis was available from 5 dogs (Table 1). Diagnoses in these dogs were marked suppurative cystitis (*n* = 4) and/or urethritis (*n* = 1), and cystitis was associated with struvite or urate urolithiasis in 1 dog each. All 5 dogs received antimicrobials and analgesics after tissue biopsy sampling. Surgical biopsies were obtained from 4 dogs (57%) during diagnostic laparotomy with cystotomy, and tissue biopsy samples were endoscopically obtained in the remaining 3 cases (43%).

*Control dogs* – Breeds included American Staffordshire terrier, Collie, Dachshund, Labrador retriever, Maltese (each *n* = 1), and mixed breed (*n* = 2). Most of these dogs (86%) had died or were euthanized for reasons unrelated to the urinary tract, and 1 dog (14%) was diagnosed with a detrusor sphincter dyssynergia and normal urinary bladder histology (but was not included in IHC analysis). All dogs (*n* = 7) were male, with 29% neutered (*n* = 2). Age-related conditions not reported to affect the urinary tract (e.g., degenerative joint disease) were not considered exclusion criteria.

Dogs with UC were significantly older (median: 11.2 years, range: 6.8–14.3 years) than dogs with non-neoplastic urinary tract inflammation (median: 6.6 years, range: 0.3–14.5 years; *P* = 0.0001) (Table 1). Body weight was not significantly different among the 3 groups of dogs (*P* = 0.5074).

**Fig. 1:**
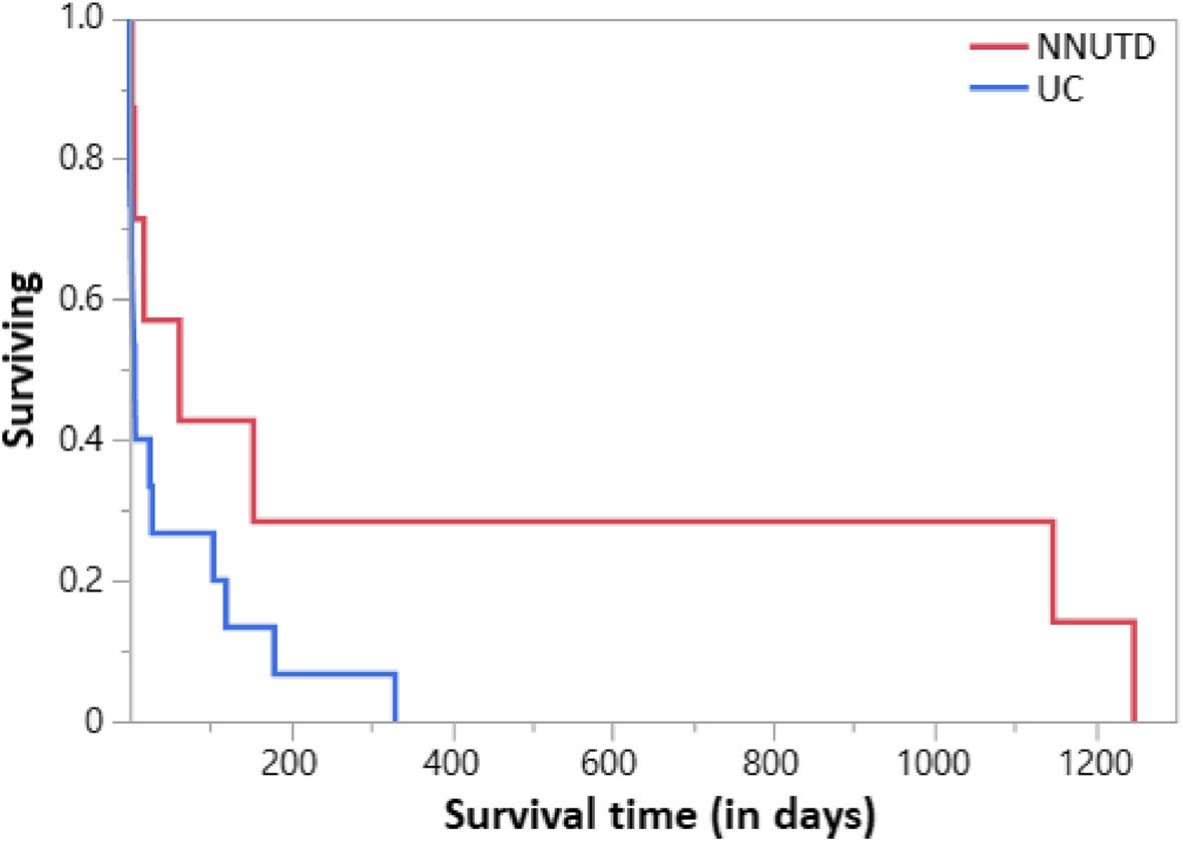
Kaplan–Meier survival plot for dogs with malignant versus benign lower urinary tract diseases. Median survival time in the urothelial carcinoma (UC) group was lower (6 days) than in dogs with non-neoplastic (inflammatory) urinary tract diseases (NNUTD; median: 60 days), but the difference was not statistically significant (*P* = 0.1067)

In the survival analysis (Fig. 1), 15 dogs in the UC group and 7 dogs in the NNUTD group reached the endpoint (death). Survival times were lower in the UC group (median: 6 days, 95% confidence interval [CI]: 0–28 days) than in the dogs with NNUTD (median: 60 days, 95%CI: 1–1,146 days), but statistical significance was not reached in the log-rank test (*P* = 0.1067).

### Tissue S100/calgranulin expression

For IHC analysis, a total of 10 tissue samples from 9 dogs diagnosed with UC were of adequate quality, 6 tissue samples from 5 dogs with NNUTD, and 11 samples from 6 control dogs (Table 2).

**Table 2:** S100A8/A9^+^ cell counts, S100A12^+^ cell counts, and the S100A8/A9^+^-to-S100A12^+^ ratio (Cal-ratio) in canine lower urinary tract tissues (*n* = 27) with a diagnosis of urothelial carcinoma or non-neoplastic urinary tract disease and in healthy controls

Parameter	Urothelial carcinoma	Non-neoplastic urinary tract disease	Healthy control dogs	*P* value^†^
Total number of tissues	10	6	11	–
urethra	5	1	5	
urinary bladder	5	5	6	
S100A8/A9^+^ cells/mm^2^	22^a^	312	21	0.3741
median [range]	[0–98]	[0–890]	[2–39]	
S100A12^+^ cells/mm^2^	**15**^**a,A**^	**75**^**A**^	**0**^**B**^	**0.0069**
median [range]	[0–69]	[0–540]	[0–7]	
Cal-ratio	**2.0**^**a**^^**,A**^	**1.3**^**A**^	**12.6**^**B**^	**0.0017**
median [range]	[1.0–11.7]	[0.5–6.6]	[1.7–40.1]	

^† ^bold font indicates significance at *P* < 0.05

^* ^values with a different capital superscript letter (A, B) differ significantly at *P* < 0.05

^a ^only available from 9 dogs

**Fig. 2:**
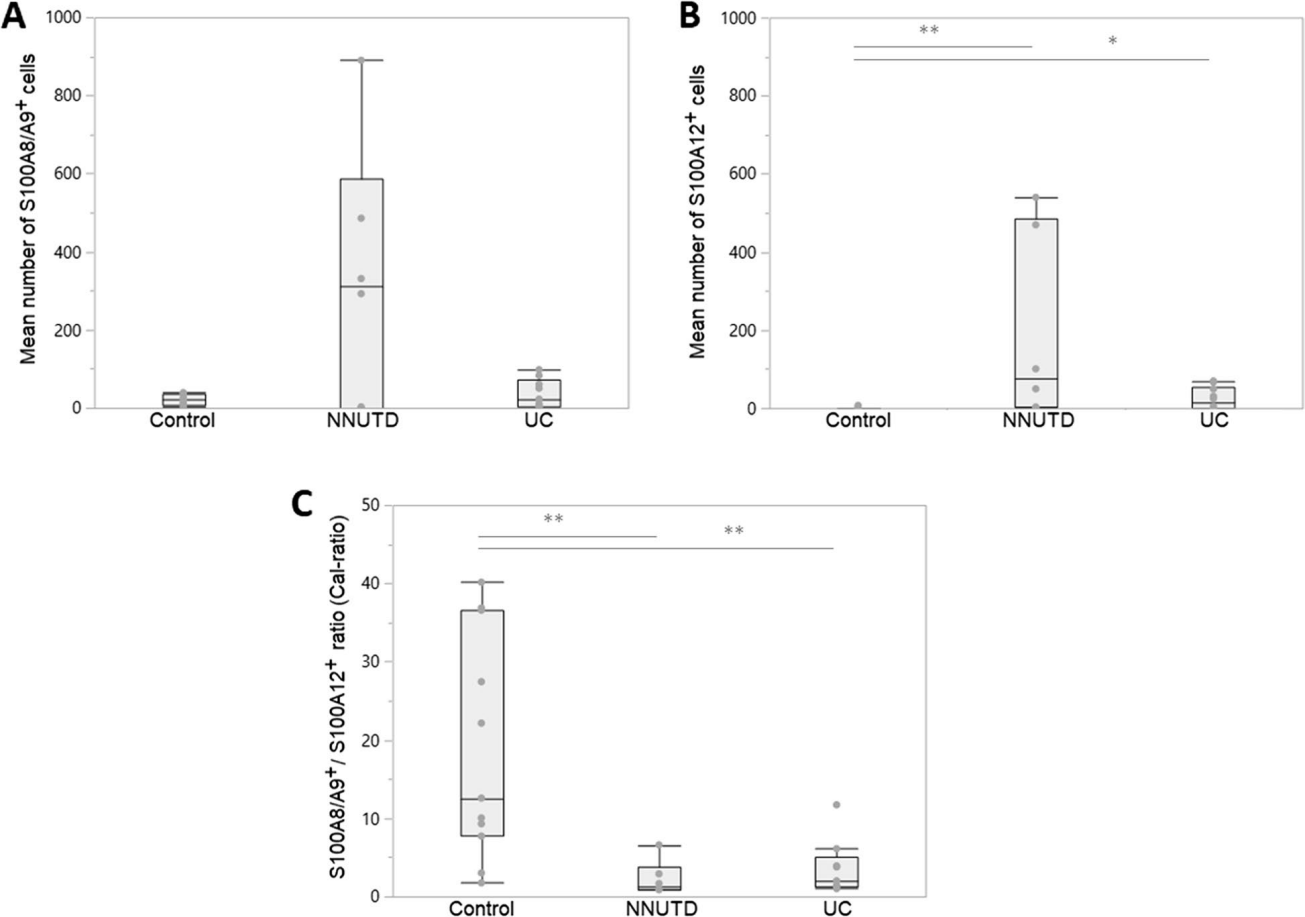
S100/calgranulin-positive cell counts in tissue biopsies of dogs with lower urinary tract diseases. **A** S100A8/A9^+^ cell counts per mm^2^ were numerically (but not statistically) higher in dogs with non-neoplastic (inflammatory) urinary tract diseases (NNUTD; median: 312, range: 0–890) than in dogs with urothelial carcinoma (UC; median: 22, range: 0–98; *P* = 0.2382) and healthy controls (control; median: 21, range: 2–39; *P* = 0.2478). **B** S100A12^+^ cell counts per mm^2^ were significantly higher in dogs with UC (median: 15, range: 0–69) and dogs with NNUTD (median: 75, range: 0–540) than in healthy controls (median: 0, range: 0–7; *P* = 0.0267 and *P* = 0.0049, respectively), but no significant difference between UC and NNUTD (*P* = 0.1372). **C** The S100A8/A9^+^-to-S100A12.^+^ ratio (Cal-ratio) was significantly lower in dogs with UC (median: 2.0, range: 1.3–4.3) and dogs with NNUTD (median: 1.3, range: 0.8–6.7) than in healthy controls (median: 12.6, range: 1.8–13.2; *P* = 0.0062 and *P* = 0.0030, respectively), but did not significantly differ between UC and NNUTD (*P* = 0.2884)

Cells staining positive for S100A8/A9 and S100A12 were predominantly infiltrating immune cells, resembling mostly neutrophils and macrophages based on their morphology, whereas negligible S100/calgranulin-staining was detected in UC cells. Numbers of S100A8/A9^+^ cells per mm^2^ were numerically smaller in dogs with UC (median: 22) compared to non-neoplastic urinary tract diseases (median: 312) but similar to the control group (median: 21) (Fig. 2). However, the difference between dogs with UC and dogs with non-neoplastic urinary tract diseases did not reach statistical significance (*P* = 0.3741). In contrast, the numbers of S100A12^+^ cells were significantly higher in dogs with UC (median: 15) and the NNUTD group (median: 75) compared to the control group (median: 0; *P* = 0.0267 and *P* = 0.0049) (Table 2, Fig. 2). However, there was no significant difference in S100A12^+^ cell counts between the UC and the NNUTD group of dogs (*P* = 0.1372). The Cal-ratio was significantly lower in UC dogs (median: 2.0) and dogs with NNUTD (median: 1.3) compared to control dogs (median: 12.6; *P* = 0.0062 and *P* = 0.0030, respectively), with no significant difference between the two disease groups (*P* = 0.2884) (Table 2, Fig. 2).

Numbers of S100A8/A9^+^ and S100A12^+^ cells were highly correlated in tissues of UC dogs (*ρ* = 0.96, *P* < 0.0001) and in dogs with non-neoplastic disease (*ρ* = 0.94, *P* = 0.0048).

### Association of S100/calgranulin expression with disease characteristics

Prior NSAID treatment or the presence of metastasis at the time of diagnosis were not found to affect the number of S100A8/A9^+^ or S100A12^+^ cells or the Cal-ratio (all *P* > 0.05). Survival time and the site evaluated (urinary bladder *vs.* urethra) or primarily affected were also not associated with the expression of S100/calgranulins or their ratio (all *P* > 0.05), albeit a trend was detected for the Cal-ratio to inversely correlate with survival time in dogs with non-neoplastic disease (ρ = -0.95, *P* = 0.0513).

Evaluating both disease groups of dogs (UC and non-neoplastic disease) combined, bacterial culture-positive dogs (*n* = 5) had higher numbers of S100A8/A9^+^ cells (median: 331/mm^2^) and S100A12^+^ cells (median: 50/mm^2^), but lower Cal-ratios (median: 1.6) than those dogs with no bacterial growth on culture (*n* = 5; medians: 11/mm^2^, 0/mm^2^, and 2.9), but the differences did not reach statistical significance (all *P* > 0.05).

### Blood neutrophil-to-lymphocyte ratio (NLR)

Dogs with non-neoplastic urinary tract disease had significantly higher neutrophil counts than those with UC (*P* < 0.0001), but no difference in lymphocyte counts between both groups (*P* = 0.7564), yielding higher NLRs in dogs with NNUTD than with UC (*P* = 0.0017) (Table 3). NLR was available for only one dog in the control group, excluding this group from NLR analysis.

**Table 3:** Neutrophil and lymphocyte counts and the neutrophil-to-lymphocyte ratio (NLR) in dogs with neoplastic or non-neoplastic urinary tract diseases (*n* = 47) and in one healthy control dog

Parameter	Urothelial carcinoma	Non-neoplastic urinary tract disease	Healthy control dogs	*P* value^†^
Total number of dogs	21	26	1	–
Neutrophil count, in 10^9^/L	**6.40**	**14.36**	5.58	**< 0.0001**
median [range]	[3.11–15.16]	[4.13–29.51]		
Lymphocyte count, in 10^9^/L	1.71	1.71	1.73	0.7564
median [range]	[0.63–3.25]	[0.32–8.05]		
NLR	**3.31**	**7.44**	3.22	**0.0017**
median [range]	[1.62–14.51]	[2.97–38.75]		

^†^ bold font indicates a significant difference between groups 1 and 2 at *P* < 0.05

The area under the ROC curve for NLR to separate dogs with UC from those with other non-neoplastic (inflammatory and/or infectious) urinary tract diseases was 0.73. Using a cut-off NLR of ≤ 2.83 yielded 41% sensitivity (95%CI: 23–61%) and a specificity of 100% (95%CI: 87–100%) to detect UC patients.

Higher NLRs showed a trend to be associated with lower numbers of tissue S100A12^+^ cells in dogs with NNUTD (*ρ* = -0.80; *P* = 0.0590).

### Association of NLR with disease characteristics

The NLR showed a moderate inverse correlation with the survival time in UC patients (*ρ* = -0.55; *P* = 0.0417), but did not distinguish between patients with UC and evidence or suspicion of metastasis at the time of diagnosis (median: 3.6) and dogs without such lesions (median: 3.3; *P* = 0.8734).

Dogs with bacterial growth on urine culture had significantly higher NLRs (median: 10.0; *n* = 10) than culture-negative dogs (median: 4.6; *n* = 19; *P* = 0.0389).

## Discussion

This study evaluated the local tissue expression of S100A8/A9 and S100A12 in neoplastic and inflammatory lower urinary tract disease in dogs compared to healthy controls. The results suggest that cells expressing these S100/calgranulin proteins – either resident or infiltrating cells – are involved in the local immune response associated with inflammatory and neoplastic disease processes in the lower urinary tract (referred to as inflammatory tumor microenvironment). This agrees with S100A12- and RAGE (receptor for advanced glycation end products)-mRNA being overexpressed in human UC [61]. Further studies should also evaluate whether the S100/calgranulin pathways (including their innate immune receptors) might determine the prognosis and/or even present potential novel therapeutic targets.

The present study followed a previous investigation by our group [49] and further evaluated the source and cellular distribution of S100/calgranulin secretion into the urine. In our previous study, urinary concentrations of S100A8/A9 and S100A12 were both significantly increased in dogs with TCC, prostatic adenocarcinoma (PCA), or UTI compared to healthy control dogs. Furthermore, the urinary Cal-ratio was significantly lower in dogs with UTI than in the other two groups of dogs, suggesting its potential as a screening test [49]. Surprisingly, the present study found no difference in S100A8/A9 positivity between dogs with UC and healthy controls, but the number of S100A8/A9^+^ cells was markedly (albeit not significantly) increased in tissues with inflammatory lesions when compared to neoplastic tissues. A possible explanation for these discrepant findings is that the sheer number of cells staining positive for S100A8/A9 and/or S100A12 does not inevitably reflect the amount of S100/calgranulin expression and release. Addressing this issue would require the quantification of S100/calgranulin staining using an automated scanning tool with a positive pixel-based algorithm [62]. Also, urinary S100/calgranulin concentrations were not measured for comparison in this present study due to the retrospective inclusion of most dogs. In people, urinary calprotectin (S100A8/A9) is increased in high-grade UC (the human counterpart of the invariably present muscle-invasive form of canine UC) compared to low-grade UC or absence of UC, provided the exclusion of renal failure and pyuria [63] because calprotectin loses specificity as a tumor marker even with sterile leukocyturia (pyuria) [65]. These findings are consistent with our numerically higher tissue S100/calgranulin-positive cell counts in dogs with (concurrent) UTI compared to dogs with no evidence of a UTI based on a quantitative urine culture.

In contrast to a previous study by our group, in which all dogs with TCC/PCA were treatment-naïve at the time of urine sampling, some dogs in the present study had received a COX-2 selective NSAIDs prior to tissue diagnosis. Thus, an effect of this treatment cannot be excluded, although prior NSAID administration was not significantly associated with different S100/calgranulin-positive cell counts. Myeloid-derived suppressor cells are attracted by inflammatory cytokines (e.g., IL-1β, IL-6, PGE_2_, and tumor growth factors), increasing their production of S100/calgranulins [65, 66]. This response is further enhanced by positive autocrine feedback mechanisms involving S100/calgranulin binding to the innate immune receptors Toll-like receptor (TLR) 4 and RAGE, resulting in increased leukocyte chemotaxis via nuclear factor (NF)-κB and MAPK signaling pathways [61, 65–67]. NF-κB increases COX-2 expression, catalyzing prostaglandin E2 (PGE_2_) synthesis [68, 69], with which COX-inhibitors aim to interfere. As a result of stopping this overt and/or perpetual process, S100/calgranulin (particularly S100A8/A9) production and release might decrease. COX-2 overexpression has been confirmed in UC cells [71, 73], and COX-2 inhibitors play an important neoadjuvant role in the treatment of UC in dogs [1, 3, 9] due to their analgesic, anti-inflammatory, and anti-tumor effects [26]. Thus, the effects of COX-inhibition on S100/calgranulin expression in canine lower urinary tract inflammatory and neoplastic diseases need to be clarified in further research.

Another possible explanation for the discrepancy between tissue S100/calgranulin expression in this study and urinary S100/calgranulin levels in the previous investigation [49] might be differences in the disease state (i.e., presence of distant and/or local metastasis) affecting S100/calgranulin expression levels. The strong trend for an inverse correlation of tissue Cal-ratios with survival time might suggest increased S100A8/A9 expression in dogs with more advanced disease. However, our study included only one dog with confirmed pulmonary metastasis, which, interestingly, was not associated with S100A8/A9^+^ or S100A12^+^ cells (data not shown). Another 3 dogs were suspected of having metastatic disease. If confirmed, the metastatic rate in our study would agree with the literature, stating that about 40% of UC patients have metastatic disease at the time of diagnosis [1, 9, 12]. Interestingly, one dog was suspected of having distant UC metastasis to the uterus, mammary gland, and lungs. UC might cause pulmonary and uterine metastases, but mammary glands are not a typical site for UC to metastasize to [12] and most dogs in the UC group were intact elderly females with a risk of developing (concurrent) primary mammary neoplasia [73, 74]. Also, to what extent other factors such as the dog’s reproductive status (most dogs were intact, whereas mostly spayed/neutered dogs were included in the previous study) might affect S100/calgranulin expression is unknown, particularly with UC being far more likely in female dogs similar to the muscle-invasive type of the disease in people. The sex distribution also differed among the groups of dogs in our study, with a female predominance in the UC group, and an effect on the results of the study cannot be excluded. To the authors’ knowledge, the possibility of sex and reproductive status affecting urinary tract S100/calgranulin expression or urine concentrations of the S100/calgranulins has not been reported. A recent study evaluating the association of clinical characteristics and lifestyle factors with S100/calgranulin concentrations in feces from healthy dogs showed a link with the reproductive status in only female dogs but no correlation with sex or reproductive status in male dogs [75]. Thus, further evaluation of a possible association between S100/calgranulin expression and canine UC biology is warranted.

Another finding was the diagnosis of UC mostly in large breed dogs (median body weight of 24 kg), which differs from other reports where UC patients had a median body weight of about 15 kg [9] and overrepresented breeds included Scottish (and other small) Terriers, Shelties, and Beagles [1, 9]. Different breed lines could explain this difference, but geographical differences linked to increased risk factors (e.g., exposure to herbicides/pesticides [4–6]) or local breed popularities might be an alternative explanation. Obesity is another risk factor for UC development [4], but reliable body condition scores were unavailable.

An unexpected finding in our study was the lack of a significant difference in survival times between dogs with UC and complete follow-up (*n* = 15; median: 6 days) than dogs with inflammatory disease (median: 60 days; *n* = 7), but only 3 dogs with UC compared to 10 dogs with NNUTD were still alive at the end of the study. Two dogs with inflammatory lower urinary tract disease were severely affected with complications and did not survive to discharge from the hospital; the third dog was euthanized due to suspicion of hemangiosarcoma. Still, survival times in dogs with UC were shorter than reported in the recent literature, likely reflecting a more conservative treatment approach elected by most owners. Most UC dogs euthanized shortly after being presented to the hospital (*n* = 5) were unable to void urine, and more invasive procedures (e.g., urethral stents, UC laser ablation) [31, 76] were declined.

This study also evaluated the diagnostic potential of the NLR in canine lower urinary tract diseases and found NLRs to be significantly higher in dogs with NNUTD, which is consistent with higher neutrophil counts and NLRs in clinically more severely affected dogs with chronic gastrointestinal inflammation [56]. Our results could be due to higher cortisol levels (e.g., systemic disease, stress) or infection causing neutrophilia (reduced endothelial adherence causing prolonged circulation times) and lymphocyte migration into affected tissues. However, the NLR increase was solely based on higher neutrophil counts, with lymphocyte counts more consistent between both disease groups. Some dogs with non-neoplastic inflammatory urinary tract disease were clinically severely affected with evidence of complications (e.g., bladder rupture causing uroabdomen and systemic inflammatory response syndrome) compared to dogs with UC. In line with this, NLR was identified as a prognostic factor in various chronic and acute inflammatory conditions in people [77, 78]. Neutrophils are involved in the response to bacterial infections, and urine cultures were positive for bacterial growth in 44% of dogs with NNUTD compared to only 25% in the UC group. However, a possible effect of prior antimicrobial treatment in some dogs cannot be completely excluded. Moderate to high sensitivity and specificity of the NLR to distinguish UC from non-neoplastic urinary tract conditions warrants further evaluation of the diagnostic value of the NLR.

We acknowledge that the retrospective inclusion of most dogs presents a limitation of this study, particularly concerning the availability of complete medical records data. However, most owners and/or primary care veterinarians were contacted for further patient and follow-up information. The study included a moderate-sized population of dogs, and an effect of treatment(s) prior to sample collection on the results in some dogs cannot be excluded. Tissue biopsies were sampled using either urethrocystoscopy or laparotomy, but relevant effects of the sampling method on the results of IHC are not to be expected given that the mucosa or mucosal side of the tissue biopsies were evaluated and significant autolysis was not present in any of the tissue samples evaluated. Lastly, different pathologists were involved in the routine histologic evaluation of the tissues, potentially introducing inter-observer variation. However, a single pathologist not involved in the initial assessments (CG) examined all tissue specimens after IHC staining, arriving at the same interpretations and diagnoses.

## Conclusions

This study confirms that the S100/calgranulins play a role in the immune response to inflammatory and neoplastic lower urinary tract diseases in dogs. However, the S100/calgranulin expression in lower urinary tract tissues detected in this study differs from our previous measurements of the same proteins in canine urine specimens. While the impact of pre-treatment (e.g., NSAIDs) and other patient factors (e.g., metastatic disease, sex, and reproductive status) on tissue S100/calprotectin expression and urinary secretion in affected dogs remains unknown, the latter might also not necessarily depend solely on the number of S100/calgranulin-expressing cells. Significant differences in S100A12^+^ cell counts and the Cal-ratio among dogs with UC or non-neoplastic inflammatory conditions and healthy controls warrant further exploration of the S100/calgranulin pathways in these diseases. NLRs were significantly higher with non-neoplastic urinary tract disease, and this routinely available marker might be a useful surrogate to distinguish UC from inflammatory conditions.

## Methods

### Ethics approval

All methods were carried out in accordance with relevant guidelines and regulations. The protocol for collection of tissue samples from dogs with urinary tract disease and healthy control dogs was independently reviewed and approved by the Regional Veterinary Council of the Free State of Saxony, Leipzig/Chemnitz, SN, Germany (TVA# 23/18), and written informed consent was obtained from all owners of dogs that were enrolled into the study.

### Sampling population

Patients (*n* = 55) for this case–control study were recruited at the Department for Small Animals, Leipzig University, College of Veterinary Medicine: dogs with UC (*n* = 22), NNUTD (*n* = 26), and healthy control dogs (*n* = 7). Inclusion criteria were the histologic documentation leading to a diagnosis of either urothelial carcinoma (UC group) or a primary or secondary inflammatory condition (e.g., cystitis, urolithiasis) where an underlying lower urinary tract neoplasm was not suspected and histologically excluded (NNUTD group). In addition, enough tissue biopsy material had to be available to perform IHC analysis and/or a complete blood cell count performed for NLR analysis. Prior treatment was not an exclusion criterion except for the administration of traditional chemotherapy or glucocorticoids (for NLR analysis).

A total of 14 dogs were prospectively enrolled, and archived tissue biopsy specimens and/or patient data were retrospectively included from 41 animals (Table 1). Of the 15 lower urinary tract disease patients included in the IHC study, 3 dogs were treatment-naïve whereas 12 dogs (86%) had received prior non-steroidal anti-inflammatory drugs (NSAID, *n* = 10; 71%), glucocorticoids (*n* = 1; 7%), and/or antimicrobials (*n* = 11; 73%) at the time of biopsy collection. Complete patient information was extracted from the electronic medical records (for retrospectively enrolled cases) and/or a study questionnaire completed by the owner and/or the attending veterinarian at patient enrolment (for prospectively enrolled cases). Dogs were followed up by contacting the referring veterinarian, owners, or both, using a standardized questionnaire. To be considered for inclusion in the control group of dogs, histopathology of urinary tract tissues had to be unremarkable.

### Tissue sample analyses

Tissue biopsies of the urinary bladder (*n* = 17) and/or urethra (*n* = 11) from dogs with UC (*n* = 9), other non-neoplastic urinary tract diseases (NNUTD; *n* = 5), and healthy controls (*n* = 6) were used for the study (Table 1).

After sampling during routine diagnostic procedures under general anesthesia (protocols optimized for the individual patient) or necropsy, the specimens were fixed in formaldehyde solution (4%), paraffin-embedded, cut into 3-µm slices, and placed on microscopy slides. Routine histopathology of urinary tract tissues was performed on hematoxylin/eosin-stained slides by one of 6 board-certified and/or nationally certified veterinary anatomic pathologists. After obtaining a histologic diagnosis, 3-µm tissue re-cuts were prepared for IHC. The specimens (3 slides of tissue samples from each dog and tissue) were deparaffinized in xylene and rehydrated in an ethanol series. After washing the slides in phosphate-buffered saline (PBS) including Tween 20 (0.025% v/v) (PBST), heat-induced antigen retrieval was performed in 0.01 M citrate buffer (pH 6.0) at 95 °C for 45 min, followed by cooling at room temperature (approximately 20 °C) for 20 min. Slides were washed twice in PBST, and the specimens were incubated for 25 min with 4% bovine serum albumin (BSA) in PBS to prevent non-specific binding. Samples were then incubated overnight at 4 °C with the primary antibody by covering one specimen each with rabbit polyclonal anti-canine S100A8/A9 (final concentration: 0.2 µg/mL) [79] or anti-canine S100A12 (final concentration: 0.25 µg/mL) [80]. A third slide was used as a negative control using normal rabbit serum (final protein concentration: 0.2 µg/mL). Slides were washed twice in PBST and then incubated with the goat anti-rabbit alkaline phosphatase-labeled secondary antibody^1^ (concentration: 1.0 µg/mL) for 60 min at room temperature. After two PBST washes, the slides were incubated with Fast Red^2^ for approximately 30 min. When optimal color had developed, the slides were first washed in PBST, followed by ddH_2_O. Mayer’s hematoxylin was used to counterstain the nuclei, with the reaction stopped after 90 s with running tap water. The slides were mounted with Fluoromount-G.^3^ After a cursory assessment by light microscopy, the slides were digitized using the Pannoramic Scan II^4^ with a 20 × objective lens. The digital images were then examined by a board-certified veterinary pathologist (CG) in a blinded fashion and using the CaseViewer digital microscopy application.^5^

**Fig. 3:**
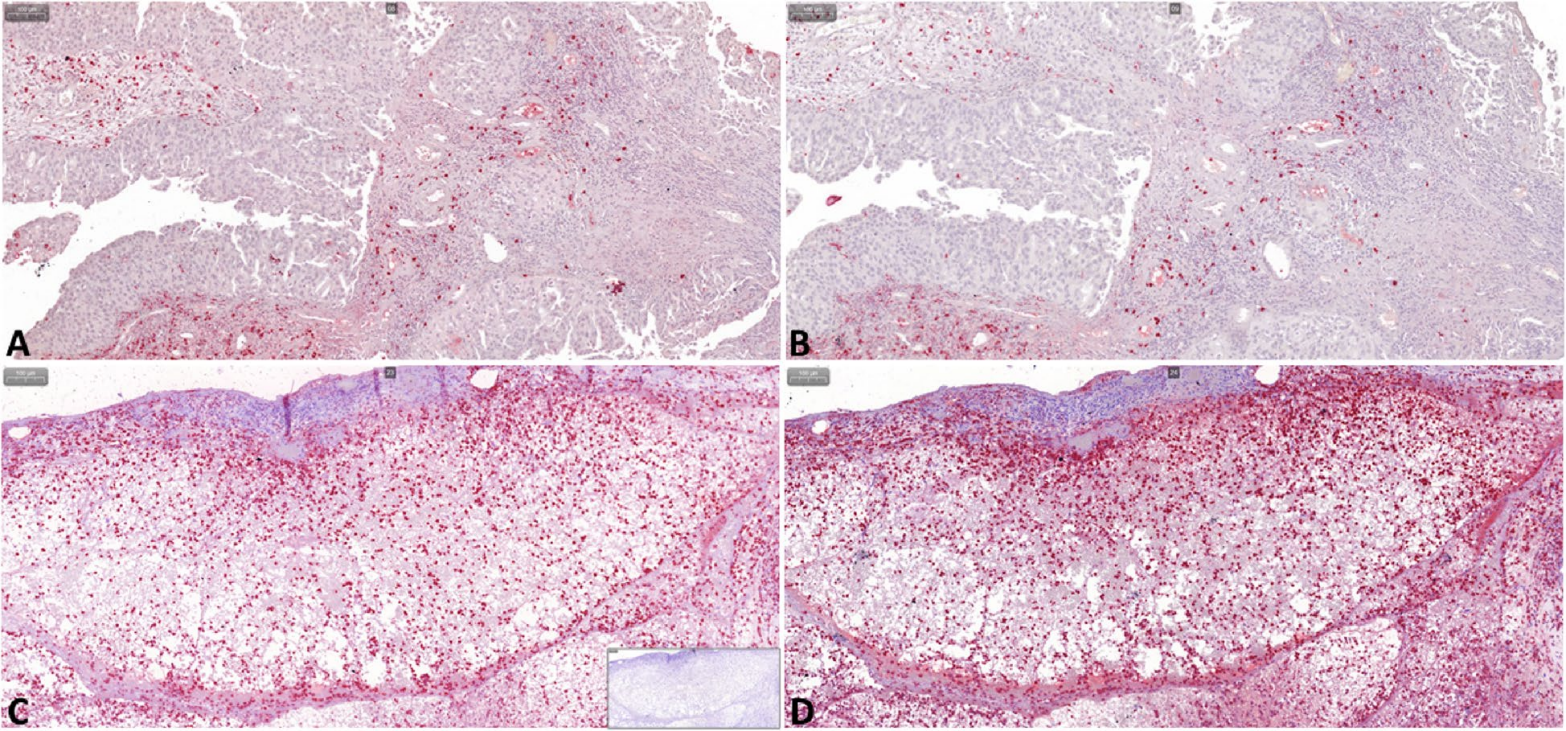
S100/calgranulin immunohistochemistry (IHC) of urinary bladder tissue biopsies in dogs with lower urinary tract disease. Upper panel: moderate numbers of infiltrating cells staining positive (Fast-red) for S100A8/A9 (**A**) or S100A12 (**B**) in a 10½-year old female mixed breed dog diagnosed with urothelial carcinoma. Lower panel: large numbers and nest-like accumulations of infiltrating cells staining positive for S100A8/A9 (**C**, insert at the bottom right: negative staining control) or S100A12 (**D**) in a 4-months old male Newfoundland dog with marked transmural suppurative cystitis. Gray scale bars at the top right corners: 100 µm

After grossly re-evaluating the histopathologic diagnosis, distribution of positive staining, and the absence of staining in the corresponding negative control (Fig. 3), 5–7 regions of 1 mm^2^ were randomly selected based on optimal tissue integrity for evaluation using CaseViewer. In these regions, all S100A8/A9-positive (S100A8/A9^+^) and S100A12-positive (S100A12^+^) cells were identified and counted if yielding a cytoplasmic or membranous signal. For data analysis, the average numbers of S100A8/A9^+^ cells and S100A12^+^ cells were calculated for all regions evaluated from the same dog, and the S100A8/A9-to-S100A12 ratio (Cal-ratio) was determined as [average number of S100A8/A9^+^ cells] / [average number of S100A12^+^ cells].

### Blood leukocyte analysis

Complete blood cell count analyses were performed using an automated blood cell analyzer. Neutrophil counts and lymphocyte counts were measured in × 10^9^ cells/L (reference intervals: 3.0–11.6 × 10^9^/L and 1.0–5.1 × 10^9^/L), and the neutrophil-to-lymphocyte ratio (NLR) was calculated as [(neutrophil count)/(lymphocyte count)] [56].

### Data analyses

A commercially available statistical software package^6^ was used for all statistical analyses. Data were tested for the assumption of normality using a Shapiro–Wilk test. Summary statistics are reported as medians and ranges (continuous data) or counts and percentages (categorical data). All parameters were tested among the 3 different groups of dogs using non-parametric multiple- (Kruskal–Wallis test: S100A8/A9^+^ cell counts, S100A12^+^ cell counts, and Cal-ratio) or two-group (Mann–Whitney *U* test: neutrophil and lymphocyte counts, NLR) comparisons. In addition, patient characteristics (age, sex, and body weight) were compared among the groups of dogs. Possible associations between numerical patient characteristics (survival time, S100/calgranulin-positive cell counts, NLR) were determined by calculating a Spearman correlation coefficient ρ. The Kaplan–Meier survival statistics was used to estimate the median (and 95% CI) time to death, and the log-rank test served to compare the survival times between dogs with UC and NNUTD. Statistical significance was set at *P* < 0.05, with *P* < 0.1 interpreted as a possible trend.

A receiver operating characteristic (ROC) curve was constructed to determine the sensitivity and specificity of the NLR to distinguish dogs with UC from dogs with other (inflammatory or infectious) urinary tract diseases, where the Youden index served to determine the best cut-off value.

## Supplementary Information

**Additional file 1:** **Supplementary Table 1.** Overview of the patient demographic, treatment, outcome, and study participation information for the dogs in-cluded in the study (*n*=55).

## Data Availability

The datasets used and/or analyzed during the current study are available from the corresponding author upon reasonable request.

## Abbreviations

AUROC: Area under the ROC curve
BSA: Bovine serum albumin
Cal-ratio: S100A8/A9-to-S100A12 ratio
COX-2: Cyclooxygenase-2
NLR: Neutrophil-to-lymphocyte ratio
NNUTD: Non-neoplastic urinary tract disease
PBS: Phosphate-buffered saline
PBST: PBS with Tween
PCA: Prostatic adenocarcinoma
ROC: Receiver operating characteristic curve
S100A8/A9: S100A8/A9 (calprotectin) protein complex
S100A12: S100A12 protein
TCC: Transitional cell carcinoma
UTI: Urinary tract infection
UC: Urothelial carcinoma
IHC: Immunohistochemistry
NSAID: Non-steroidal anti-inflammatory drug

## Footnotes

1 Goat anti-rabbit alkaline phosphatase-labeled immunoglobulin, Dianova, Hamburg, Germany.

2 SigmaFast™ Fast Red TR/Naphthol AS, Merck, Darmstadt, Germany.

3 Fluoromount-G™ Mounting Medium, Thermo Fisher, Dreieich, Germany.

4 Pannoramic Scan II, 3D-Histech Ltd., Budapest, Hungary.

5 CaseViewer digital microscopy application, Slides.3D-Histech Ltd., Budapest, Hungary.

6 JMP v13.0, SAS Institute Inc., Cary, NC, USA.
