# Supplementary material for: Tissue S100/calgranulin expression and blood neutrophil-to-lymphocyte ratio (NLR) in dogs with lower urinary tract urothelial carcinoma
Source: BMC Vet Res. 2022 Nov 21;18:412. doi: 10.1186/s12917-022-03513-z (PMC9680134; doi:10.1186/s12917-022-03513-z)
Supplement: Supplementary file 1 — Additional file 1: Supplementary Table 1. Overview of the patient demographic, treatment, outcome, and study participation information for the dogs in-cluded in the study (n=55). [file 12917_2022_3513_MOESM1_ESM.docx]

**Supplementary Table 1 – Overview of the patient demographic, treatment, outcome, and study participation information for the dogs included in the study (n=55).**

| **No.** | **Breed** | **Sex** | **Neuter status** | **Age (in years)** | **Body weight (in kg)** | **Study inclusion** | **UTI** | **Prior NSAID** | **Biopsy type** | | **Tissue evaluated** | | **Diagnosis** | | **Evidence of metastasis** | | **Treatment** | | **Survival time (in days)** | | **Study parts** | | **Outcome** | |
| --- | --- | --- | --- | --- | --- | --- | --- | --- | --- | --- | --- | --- | --- | --- | --- | --- | --- | --- | --- | --- | --- | --- | --- | --- |
| 1 | Mixed breed | female | intact | 10.5 | 25 | prospective | *nc* | no | surgical (postmortem) | | urinary bladder, urethra | | UC | | lung | | none | | 0 | | IHC, NLR | | euthanized/died | |
| 2 | Cocker spaniel | female | intact | 12.8 | 16.3 | retrospective | *nc* | no | endoscopic | | urinary bladder | | UC | | no | | *nd* | |  | | IHC, NLR | | lost to follow-up | |
| 3 | Mixed breed | female | intact | 10.5 | 6.2 | retrospective | no | meloxicam | endoscopic | | urinary bladder | | UC | | no | | metronomic chlorambucil, meloxicam | | 180 | | IHC, NLR | | euthanized/died | |
| 4 | Soft-Coated Wheaton terrier | female | spayed | 11.6 | 19.0 | retrospective | *nc* | meloxicam | endoscopic | | urethra | | UC | | no | | none | | 0 | | IHC, NLR | | euthanized/died | |
| 5 | Bernese Mountain dog | female | intact | 9.0 | 48.0 | retrospective | *nc* | meloxicam | endoscopic | | urinary bladder | | UC | | no | | analgesia | | 120 | | IHC, NLR | | euthanized/died | |
| 6 | Mixed breed | female | intact | 11.7 | 31.0 | retrospective | yes (*E. coli*) | meloxicam | endoscopic | | urethra | | UC | | spleen (suspected) | | antibiotic, analgesia, alfuzosine | | 330 | | IHC | | euthanized/died | |
| 7 | Labrador retriever | female | intact | 9.9 | 27.8 | prospective | *nc* | robenacoxib | endoscopic | | urinary bladder | | UC | | spleen (suspected) | | analgesia | | 3 | | IHC, NLR | | euthanized/died | |
| 8 | Mixed breed | female | intact | 9.7 | 30.0 | prospective | *nc* | meloxicam | endoscopic | | urethra | | UC | | lung, mamma, uterus (suspected) | | none | | 2 | | IHC, NLR | | euthanized/died | |
| 9 | Border collie | female | spayed | 13.0 | 22.9 | prospective | no | carprofen | endoscopic | | urethra | | UC | | no | | none | | 7 | | IHC, NLR | | euthanized/died | |
| 10 | West Highland White terrier | female | intact | 9.8 | 7.5 | prospective | no | no | endoscopic | | urinary bladder | | UC | | no | | analgesia | |  | | IHC, NLR | | alive | |
| 11 | Mixed breed | male | neutered | 14.3 | 26.0 | retrospective | *nc* | no | –– | | urinary bladder | | UC | | sublumbar lymph nodes (suspected) | | chemotherapy (mitoxantron) | |  | | NLR | | lost to follow-up | |
| 12 | Mixed breed | female | spayed | 11.7 | 30.5 | retrospective | yes (*S. intermedius*) | meloxicam | –– | | urinary bladder | | UC | | no | | none | | 0 | | NLR | | euthanized/died | |
| 13 | Labrador retriever | female | intact | 12.0 | 34 | retrospective | no | firocoxib | –– | | urinary bladder | | UC | | no | | analgesia (meloxicam) | |  | | NLR | | lost to follow-up | |
| 14 | Bordeaux mastiff | female | spayed | 7.0 | 49.0 | retrospective | *nc* | no | –– | | urinary bladder | | UC | | no | | chemotherapy (mitoxantron) | | 25 | | NLR | | euthanized/died | |
| 15 | Mixed breed | male | neutered | 12.0 | 27.0 | retrospective | no | no | –– | | urinary bladder | | UC | | no | | analgesia (carprofen) | | 6 | | NLR | | euthanized/died | |
| 16 | Eurasier | male | intact | 12.0 | 26.0 | retrospective | no | no | –– | | urinary bladder | | UC | | no | | analgesia (meloxicam) | | 1 | | NLR | | euthanized/died | |
| 17 | Labrador retriever | female | spayed | 13.0 | 35.3 | retrospective | no | no | –– | | urinary bladder | | UC | | no | | analgesia (meloxicam) | | 104 | | NLR | | euthanized/died | |
| 18 | Jack Russell terrier | female | spayed | 9.6 | 9.1 | retrospective | no | meloxicam | –– | | urinary bladder | | UC | | no | | chemotherapy (mitoxantron) | |  | | NLR | | lost to follow-up | |
| 19 | Mixed breed | female | intact | 13.1 | 8.4 | retrospective | *nc* | meloxicam | –– | | urinary bladder | | UC | | no | | chemotherapy (mitoxantron) | |  | | NLR | | alive | |
| 20 | Airedale terrier | male | intact | 8.0 | 31.0 | retrospective | *nc* | meloxicam | –– | | urinary bladder | | UC | | no | | analgesia (meloxicam) | |  | | NLR | | alive | |
| 21 | Mixed breed | female | intact | 6.8 | 25.0 | retrospective | no | meloxicam | –– | | urinary bladder | | UC | | no | | analgesia (meloxicam) | | 28 | | NLR | | euthanized/died | |
| 22 | Mixed breed | female | intact | 10.8 | 26.0 | retrospective | yes (*E. coli*) | meloxicam | –– | | urinary bladder | | UC | | no | | analgesia (meloxicam) | | 5 | | NLR | | euthanized/died | |
| 23 | Labrador retriever | female | intact | 8.8 | 30.0 | retrospective | yes (*Enterobacter cloacae*) | no | endoscopic | | urinary bladder | | NNUTD | | –– | | *nd* | | 4 | | IHC, NLR | | euthanized/died | |
| 24 | Labrador retriever | female | intact | 10.7 | 37.0 | retrospective | no | meloxicam | endoscopic | | urethra | | NNUTD | | –– | | *nd* | |  | | IHC, NLR | | lost to follow-up | |
| 25 | Newfoundland | male | intact | 0.3 | 14.5 | retrospective | yes (*P. mirabilis*, *S. intermedius*) | no | surgical | | urinary bladder | | NNUTD | | –– | | cystotomy, antibiotic, analgesia | | 16 | | IHC, NLR | | euthanized/died | |
| 26 | Mixed breed | male | neutered | 6.0 | 35.0 | retrospective | yes (*S. intermedius*) | meloxicam | surgical | | urinary bladder | | NNUTD | | –– | | *nd* | |  | | IHC, NLR | | alive | |
| 27 | Dalmatiner | male | intact | 6.2 | 35.0 | retrospective | no | no | endoscopic | | urinary bladder | | NNUTD | | –– | | *nd* | |  | | IHC, NLR | | lost to follow-up | |
| 28 | Shih tzu | male | neutered | 2.7 | 5.5 | prospective | yes (*S. intermedius*) | no | surgical | | urinary bladder | | NNUTD | | –– | | cystotomy, antibiotic, analgesia, intermittent catheterization | |  | | IHC, NLR | | alive | |
| 29 | Mixed breed | male | intact | 11.7 | 54.0 | prospective | no | robenacoxib | surgical | | urinary bladder | | NNUTD | | –– | | cystotomy | | 60 | | IHC, NLR | | euthanized/died | |
| 30 | Doberman | male | neutered | 13.0 | 34.0 | retrospective | *nc* | meloxicam | –– | | urinary bladder | | NNUTD | | –– | | *nd* | |  | | NLR | | lost to follow-up | |
| 31 | Yorkshire terrier | male | intact | 8.6 | 3.0 | retrospective | *nc* | *unknown* | –– | | urinary bladder | | NNUTD | | –– | | *nd* | |  | | NLR | | lost to follow-up | |
| 32 | Pug | female | spayed | 5.7 | 8.5 | retrospective | *nc* | no | –– | | urinary bladder | | NNUTD | | –– | | *nd* | |  | | NLR | | lost to follow-up | |
| 33 | Schnauzer | male | intact | 14.5 | 16.7 | retrospective | yes (*E. coli*) | *unknown* | | –– | | urinary bladder | | NNUTD | | –– | | *nd* | |  | | NLR | | lost to follow-up |
| 34 | Beagle | male | intact | *nd* | *nd* | retrospective | no | no | –– | | urinary bladder | | NNUTD | | –– | | *nd* | | 152 | | NLR | | euthanized/died | |
| 35 | Dachshund | female | spayed | 4.6 | 4.8 | retrospective | no | no | –– | | urinary bladder | | NNUTD | | –– | | *nd* | |  | | NLR | | alive | |
| 36 | Berger de Brie | male | neutered | 6.6 | 53.5 | retrospective | *nc* | no | –– | | urinary bladder | | NNUTD | | –– | | *nd* | |  | | NLR | | alive | |
| 37 | German Shepherd dog | male | intact | 10.7 | 34.0 | retrospective | yes (*S. intermedius*) | no | –– | | urinary bladder | | NNUTD | | –– | | *nd* | | 1 | | NLR | | euthanized/died | |
| 38 | Yorkshire terrier | male | intact | 6.8 | 6.7 | retrospective | *nc* | no | –– | | urinary bladder | | NNUTD | | –– | | *nd* | |  | | NLR | | alive | |
| 39 | Rottweiler | male | intact | 5.5 | 42.0 | retrospective | *nc* | no | –– | | urinary bladder | | NNUTD | | –– | | *nd* | |  | | NLR | | lost to follow-up | |
| 40 | Schnauzer | male | intact | 5.8 | 8.0 | retrospective | yes (*S. canis*) | no | –– | | urinary bladder | | NNUTD | | –– | | *nd* | | 1146 | | NLR | | euthanized/died | |
| 41 | Yorkshire terrier | male | intact | 7.0 | 3.8 | retrospective | *nc* | no | –– | | urinary bladder | | NNUTD | | –– | | *nd* | | 1247 | | NLR | | euthanized/died | |
| 42 | Bernese Mountain dog | male | intact | 8.0 | 48.8 | retrospective | yes (*S. intermedius*) | no | –– | | urinary bladder | | NNUTD | | –– | | *nd* | |  | | NLR | | lost to follow-up | |
| 43 | Chihuahua | male | intact | 4.8 | 5.9 | retrospective | no | no | –– | | urinary bladder | | NNUTD | | –– | | *nd* | |  | | NLR | | alive | |
| 44 | Mixed breed | female | spayed | 5.9 | 11.0 | retrospective | no | no | –– | | urinary bladder | | NNUTD | | –– | | *nd* | |  | | NLR | | alive | |
| 45 | Mixed breed | male | neutered | 4.2 | 11.6 | retrospective | no | no | –– | | urinary bladder | | NNUTD | | –– | | *nd* | |  | | NLR | | alive | |
| 46 | Mixed breed | female | spayed | 6.8 | 5.2 | retrospective | no | no | –– | | urinary bladder | | NNUTD | | –– | | *nd* | |  | | NLR | | alive | |
| 47 | Yorkshire terrier | male | intact | 11.4 | 5.0 | retrospective | *nc* | no | –– | | urinary bladder | | NNUTD | | –– | | *nd* | |  | | NLR | | alive | |
| 48 | English bulldog | male | intact | 3.3 | 25.0 | retrospective | no | meloxicam | –– | | urinary bladder | | NNUTD | | –– | | *nd* | |  | | NLR | | alive | |
| 49 | Labrador retriever | male | neutered | 9.8 | 41.5 | prospective | no | no | endoscopic | | urinary bladder | | Control | | –– | | –– | | –– | | NLR | | –– | |
| 50 | Mixed breed | male | intact | 4.0 | 19.2 | prospective | no | *unknown* | surgical (post mortem) | | urinary bladder, urethra | | Control | | –– | | –– | | –– | | IHC | | –– | |
| 51 | American Stafford-shire terrier | male | intact | 2.0 | 26. | prospective | no | *unknown* | surgical (post mortem) | | urinary bladder, urethra | | Control | | –– | | –– | | –– | | IHC | | –– | |
| 52 | Collie | male | intact | 10.0 | 21.8 | prospective | no | *unknown* | surgical (post mortem) | | urinary bladder | | Control | | –– | | –– | | –– | | IHC | | –– | |
| 53 | Maltese | male | neutered | *nd* | 7.7 | prospective | no | *unknown* | surgical (post mortem) | | urinary bladder, urethra | | Control | | –– | | –– | | –– | | IHC | | –– | |
| 54 | Dachshund | male | intact | 8.0 | 5.2 | prospective | no | *unknown* | surgical (post mortem) | | urinary bladder, urethra | | Control | | –– | | –– | | –– | | IHC | | –– | |
| 55 | Mixed breed | male | intact | 5.0 | 27.0 | prospective | no | *unknown* | surgical (post mortem) | | urinary bladder, urethra | | Control | | –– | | –– | | –– | | IHC | | –– | |
| IHC: immunohistochemistry; *nc*: not cultured; *nd*: not documented; NLR: neutrophil-to-lymphocyte ratio; NNUTD: non-neoplastic urinary tract disease; NSAID: non-steroidal anti-inflammatory drug; UC: urothelial carcinoma; UTI: urinary tract infection. | | | | | | | | | | | | | | | | | | | | | | | | |
